# Quantum Statistical Behaviors of Carriers in Strong Inversion Layers Associated with Mobility and Threshold Voltage in FinFET Transistors

**DOI:** 10.3390/mi17070808

**Published:** 2026-07-02

**Authors:** Hsin-Chia Yang, Sung-Ching Chi, Han-Ya Yang

**Affiliations:** 1 Department of Electronic Engineering, Ming Hsin University of Science and Technology, Hsinchu County 30401, Taiwan; chisc@must.edu.tw; 2 Department of Electronic Physics, National Yang-Ming Chiao-Tung University, Hsinchu City 300, Taiwan; rebeccaxd29@yahoo.com.tw

**Keywords:** MOSFET, FinFET, negative threshold voltage, radiation by accelerated charges, strong inversion layer, kink effects

## Abstract

Gated transistors in the form of MOSFET, FinFET, or IGBT are capable of controlling and transferring either signals or powers. These capabilities are closely associated with applied biases on Gates, which surpass the respective threshold voltages. Source/Drain bias, V_DS_, then establishes the electric field, E_DS_, driving carriers to flow with a speed which is proportional to E_DS_ with the proportionality, termed mobility, μ. The mobility somewhat addresses the electrical performances of the specific transistor, and is V_GS_-dependent, where the generated electric field is perpendicular to the interface in between the Gate and the Gate oxide and is directed across the channel. The mobility may be treated as the collective quantum statistical behaviors of carriers, i.e., electrons or fermions. It is worth analyzing the electrical performances by way of quantum statistics. Nevertheless, the threshold voltages are surprisingly negative on FinFETs as the fitting is performed, which means that I_DS_ would flow even without applied voltage on the Gate. I_DS_-V_DS_ characteristic curves with negative threshold voltage intriguingly perform just like the other ones with positive threshold voltages. Therefore, there might exist some kind of mechanism enhancing strong inversion layers that is responsible for the characteristics. In this paper, characteristic curves of FinFETs may be well fitted by using both modified characteristic formulas and the proposed kink effects. The extracted parameters (k_N_, Vth, λ) thus provide information on mobility, concentration of p (1/cm^3^), or even leakage current. Also, the mobility, μ, here is analyzed by using Fermion statistics. Furthermore, trivial solutions for the specific boundary conditions, V_GS_ = 0 V, surrounding the channel are presented, where one of the possibilities proposed is the mass plasma oscillation of electrons, which might be an option for addressing the negative threshold voltage.

## 1. Introduction

### 1.1. Preparation of FinFET Devices

**Figure 1:**
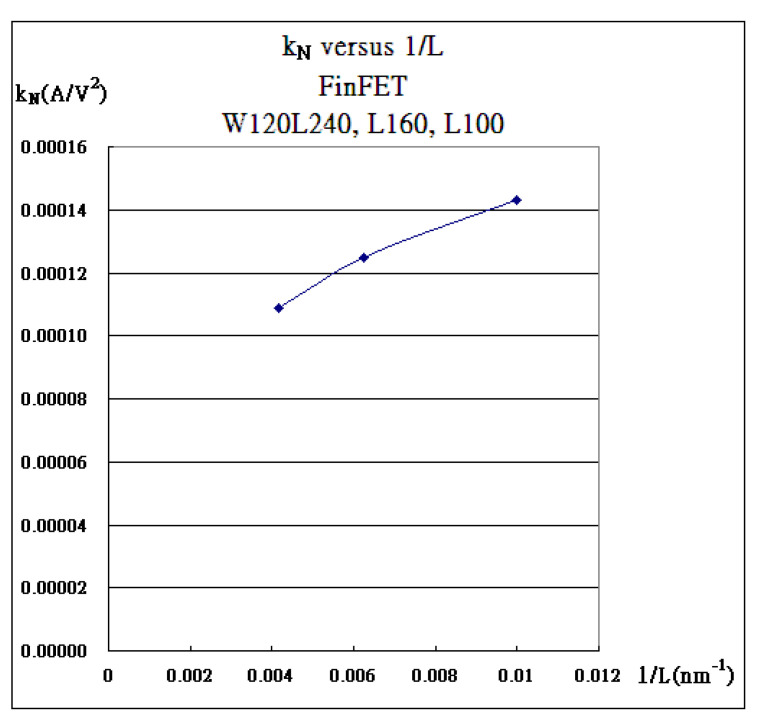
k_N_ is supposed to be proportional to 1/L.

By ionic dry etching the epi-silicon layer which is pregrown by flowing silane (SiH_4_) accompanied with slightly adultered HCl, three sizes of fin width (110 nm, 115 nm, 120 nm) of 3D “I” are formed with aspect ratios of 9, with the ends named the Source and Drain. The epi-silicon fin channel is grown with the ultra-thin gate oxide of 14 angstroms and covered with arsenic heavily doped poly-silicon. After SF_6_ dry poly-silicon etching, various channel lengths (e.g., 240 nm, 160 nm, 120 nm, 100 nm) are characterized with probe-testing on I_DS_-V_DS_ [1,2,3]. FinFETs with 110 nm fin widths are ruled out because of flattened and suppressed characteristic curves in the triode region, which are not acceptably normal FETs. FinFETs with 115 nm fin widths are not feasible because of non-linear dependence of k_N_ verse the inverse of channel length [4,5,6,7]. The only reliable transistors are those with channel fin widths of 120 nm, which demonstrate the feasibility and set up the design rule in Mask Layout referred to in reference [1]. k_N_ is linearly dependent of (1/L) with a fin width of 120 nm, where three FinFETs (W120L240, W120L160, and W120L100) are analyzed in the following graph, as shown in Figure 1.

### 1.2. A Conclusive Model Addressing Characteristic Curves of Field-Effect Transistors

A newly generated model addressing characteristics of field-effect transistors (FETs) is due to a little modification over the conventional formulas, which extract the mobility (μ) from the integration over the channel variable from 0 to V_DS_ at constant V_GS_ [8]. The characteristic curves always become saturated at V_DS_ = (V_GS_ − Vth) and have an almost straight saturation line with the slope of λ. With the determined Vth and λ, k_N_ can be easily found and a discrepancy always occurs between the as-measured curve and the as-formulated one. The discrepancy is easily eliminated by introducing a Gaussian-like kink effect such that as-measured curves are well fitted. This conclusive model is presented in Equation (1) as follows:

(1) $$\left\{\begin{array}{l} I_{DS}(triode)=k_{N}[(V_{GS}-V_{th})V_{DS}-\frac{V_{DS}^{2}}{2}](1+\lambda V_{DS})\\ -I_{kink}\exp[-W_{kink}{\left(V_{DS}-V_{kink}\right)}^{2}]\\ I_{DS}(saturation)=k_{N}[\frac{{\left(V_{GS}-V_{th}\right)}^{2}}{2}](1+\lambda V_{DS})\\ -I_{kink}\exp[-W_{kink}{\left(V_{DS}-V_{kink}\right)}^{2}] \end{array}\right.$$

where

$$\begin{array}{l} k_{N}=\frac{C_{ox}^{\left(1\right)}W_{eff}\mu_{N}}{L_{o}},\\ V_{th}=(-0.56-\left|\Phi_{p}\right|{)}_{FB}-\frac{Q_{ox}^{\prime }}{C_{ox}^{\prime }}+2\left|\Phi_{p}\right|+\frac{Q_{dep}^{\prime }}{C_{ox}^{\prime }}\\ with\\ V_{GS}\geq 2\left|\Phi_{p}\right|=2\frac{k_{B}T}{e}\ln(\frac{p}{n_{i}})(\mathrm{deep}\mathrm{strong}\mathrm{inversion}), \end{array}$$

and fitting by minimizing root-mean-square formulas as follows:

$$\delta_{rms}=\sqrt{\frac{\sum_{i=1}^{N}{\left(I_{DS\_fitting}-I_{DS\_as-measured}\right)}^{2}}{N}}$$

and (V_kink_, I_kink_) is the maximum discrepancy with W_kink_ for the effective Gaussian width. Normally, concerning non-zero bias, the model is applied to the Gate such that the channel is first depleted and then strongly inversed. The strong inversion layer is responsible for conduction of current in between the Source and Drain [9,10,11,12].

### 1.3. Potential Energy Associated with Depletion Region and Strong Inversion Layer

By solving the divergence of electric field in Maxwell’s equations, the electrical potential energy as applied with a Gate bias (V_GS_) is expressed as follows:

$$U(x)=-eV(x)=-\frac{pe^{2}}{2\varepsilon_{Si}}{\left(x-D\right)}^{2}+\frac{pe^{2}}{2\varepsilon_{Si}}D^{2}-eV_{GS}.$$

with boundary conditions

$$\left\{\begin{array}{l} U(0)=-eV_{GS}\\ U(D)=0 \end{array}\right..$$

**Figure 2:**
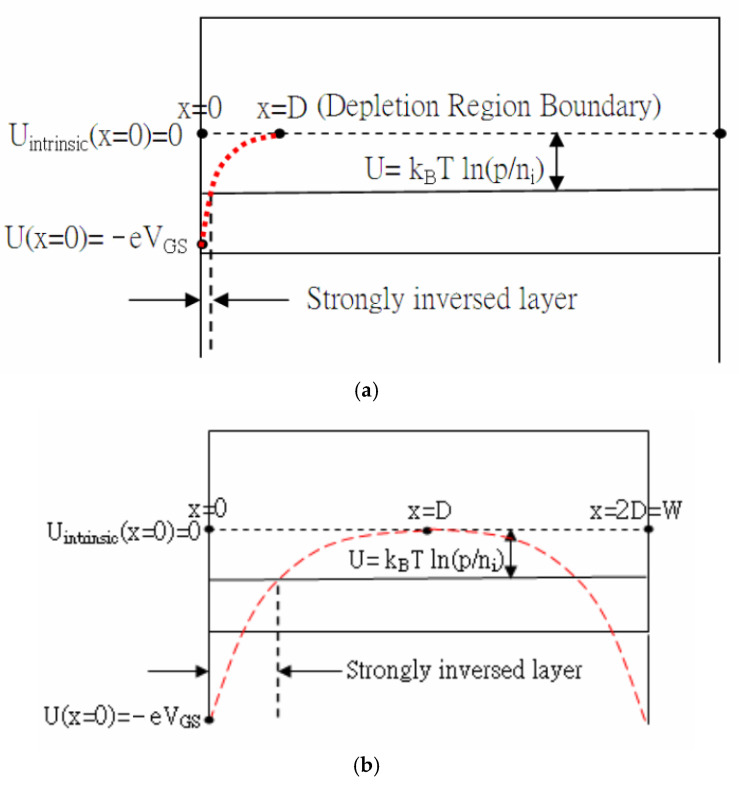
Red detached lines indicate the potential energy. (**a**) Constant p-type Fermi energy and silicon energy gap with intrinsic Fermi energy that may bend down as positive bias is applied to the Gate. (**b**) FinFETs (W120L100, W120L160, and W120L240) are supposed to have the fin width 2D = 120 nm at V_GS_ = 1 V, which corresponds to p(1/cm^3^) = 3.7 × 10^17^/cm^3^.

**Figure 3:**
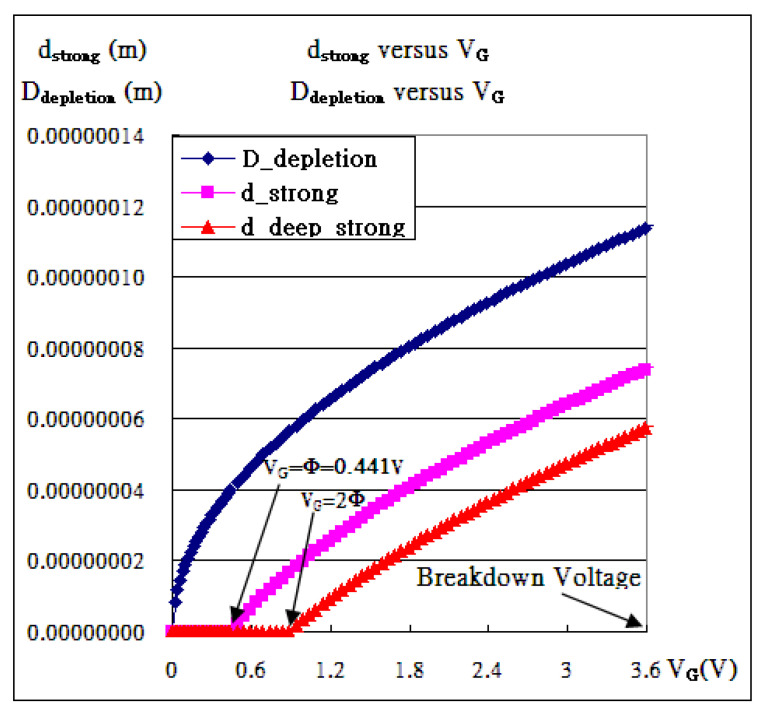
Depletion region and strong inversion layer at non-zero V_GS_, and breakdown voltage (3.6 V) along channel length with p(1/cm^3^) = 3.7 × 10^17^/m^3^.

The thickness of depletion region, $D=\sqrt{\frac{2\varepsilon_{Si}}{pe}V_{GS}}$, is to be derived as the boundary condition U(D) = 0 is applied. Also, the thickness of the strong inversion layer is to be resolved by setting U(d_Φ_) = −k_B_T ln(p/n_i_). Graphs showing the depletion region and the strong inversion layer are presented in Figure 2 and Figure 3. In addition, the effective width of the FinFET is set to 19 times the thickness of the depletion region at V_GS_ = 1.0 V, and the concentration of the channel is thus identified as 3.7 × 10^17^ (1/cm^3^), whose breakdown voltage of the channel is 3.6 V [10].

(2) $$\begin{array}{l} \left\{\begin{array}{l} d_{\Phi }=x_{S\_\Phi }=D_{dep}(1-\sqrt{\frac{\Phi }{V_{GS}}})\\ d_{2\Phi }=x_{S\_2\Phi }=D_{dep}(1-\sqrt{\frac{2\Phi }{V_{GS}}}) \end{array}\right. \end{array}$$

### 1.4. NFinFETs with Negative Threshold Voltages

An idea arises regarding the semiconductor that there are negative threshold voltages being applied to n-channel transistors. That is, transistors without strong inversion layers within deplete regions can even become conductive at V_GS_ = 0.0 V as non-zero V_DS_ is applied to the Source and Drain. Intriguingly at V_GS_ = 0.0 V, I_DS_-V_DS_ characteristic curves with negative Vth look like the others with positive Vth, which have the features of characteristic curves containing a triode region and saturation region. Zero V_GS_ is thought not to build up a depletion region or even a strong inversion layer. Meanwhile, a depletion region helps contribute positive quantity to threshold voltage. An example is shown in Figure 2. But why is the channel conductive and why does the Drain/Source current reach satisfaction at *V_DS_* = (*V_GS_* − *Vth*). The explanation might be quantum effects or something else. An idea came from the bulk or collective plasma oscillation making instantaneous positive and negative biases on either side of the slim channel of the FinFET, which cause an instantaneous strong inversion layer on either side of the fin channel based on the plasma frequency. This creates electromagnetic wave radiation from all FinFETs in IC, even though its intensity might be weak due to non-coherent motions. Nevertheless, characteristic curves are demanded to fit the modified conventional formulas (one is in the triode region and the other in the saturation region).

Solving the field solution, V(x, y, z), is important for addressing the whole field effects inside the transistor. The uniqueness theorem may provide solid assurance of solutions with fixed boundary conditions, which may give information about how the transistor has negative threshold voltage if the applied Gate bias is zero with non-zero V_DS_.

### 1.5. Statistical Behaviors of Carriers

There are two kinds of carriers, electrons and holes, in a semiconductor. Both carry either electrical power or electrical signals, which statistically need large quantities. Intriguingly, they are called “Fermions” and ought somewhat to obey a certain statistical regulation, Fermi–Dirac statistics, which requires the order of trillion times trillion to make it effective at lower temperature. In principle, no two identical fermions are allowed to exist in the same state [13]. Fermi-Dirac Statistics with *p_o_ =* 3.7 × 10^17^/cm^3^ whose Fermi-energy is U(d) is as follows:

$$\begin{array}{l} p(x)=\frac{p_{o}}{e^{\frac{u}{k_{B}T}}+1}\\ and\\ p(x)=\frac{p_{o}}{e^{\frac{U(x)-U(d)}{k_{B}T}}+1}=\frac{p_{o}}{e^{\frac{-e[V(x)-V(d)]}{k_{B}T}}+1}\\ \mathrm{For}e[V(x)-V(d)]<<k_{B}T\\ p(x)\overset{T=300K}{\to }\frac{p_{o}}{e^{\frac{-[V(x)-V(d)]}{0.0259}}+1}\\ p(x)\to p_{o}\frac{V(x)-V(d)}{0.0259}\leq 0.1p_{o} \end{array}$$

Their statistical behavior may approach Maxwell–Boltzmann statistics if the temperature is above 1000 K. As the ambient temperature in the circumstances is at room temperature corresponding to (k_B_T/e) = 0.0259 V, the voltage difference, (V(x) − V(d)), around the strong inversion layer may be reasonably less than one tenth of 0.0259 V. CMOSFET, CFinFET, and CGAAFET mainly control or convey signals and thus have collective carriers move around according to some specific circuit design. Those collective behaviors of carriers may explain I–V characteristic curves of transistors, kink effects, mobility, etc.

### 1.6. Mobility

Collective carriers move along the Source to the Drain depending on the transverse electric field (E). The stronger E is, the lower the mobility (μ) is, which is reasonably explained as attracted carriers hitting the interfacial oxide and causing retardation. For planar MOSFET, FinFET, and GAAFET, E is actually proportional to (V_GS_ − Vth) at non-zero V_GS_. Nevertheless, it still does not address how the mobility depends on the electric field as V_GS_ = 0 V.

In this paper, three FinFET transistors (W120L100, W120L160, and W120L240) at various applied Gate biases are fitted with three parameters (k_N_, Vth, λ), followed and finalized by kink effects. Mobility is statistically analyzed and discussed. Fitting of characteristic curves of three kinds of transistors at V_GS_ = 0 V is feasibly presented. Laplace’s differential equation with boundary conditions at V_GS_ = 0 V is solved, and the solution, V(x,y,z), resulting in plasma oscillation being introduced physically, addressing the negative threshold voltage.

## 2. Characteristics Curves Fitted with Kink Effects

### 2.1. Extraction of Three Parameters (k_N_, Vth, λ) at Non-Zero-Biased V_GS_

**Figure 4:**
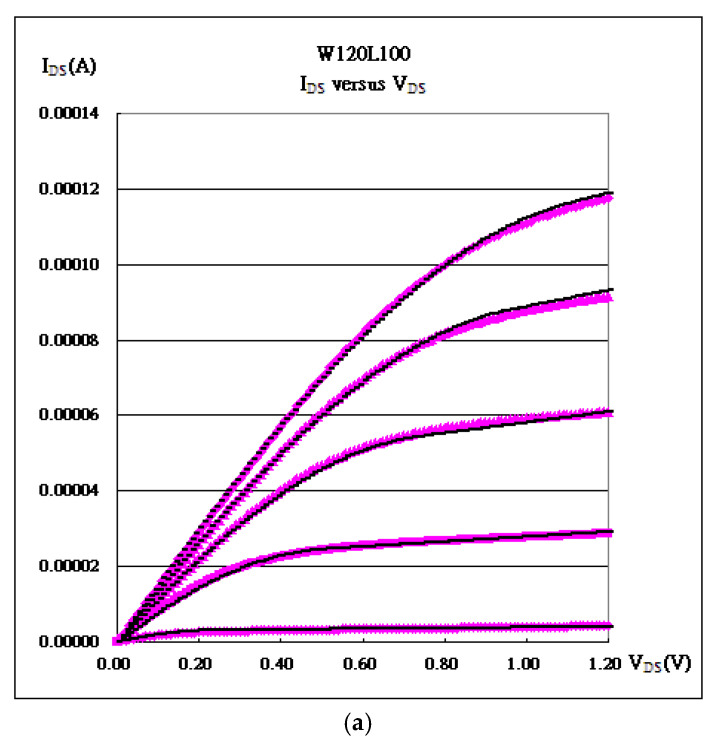

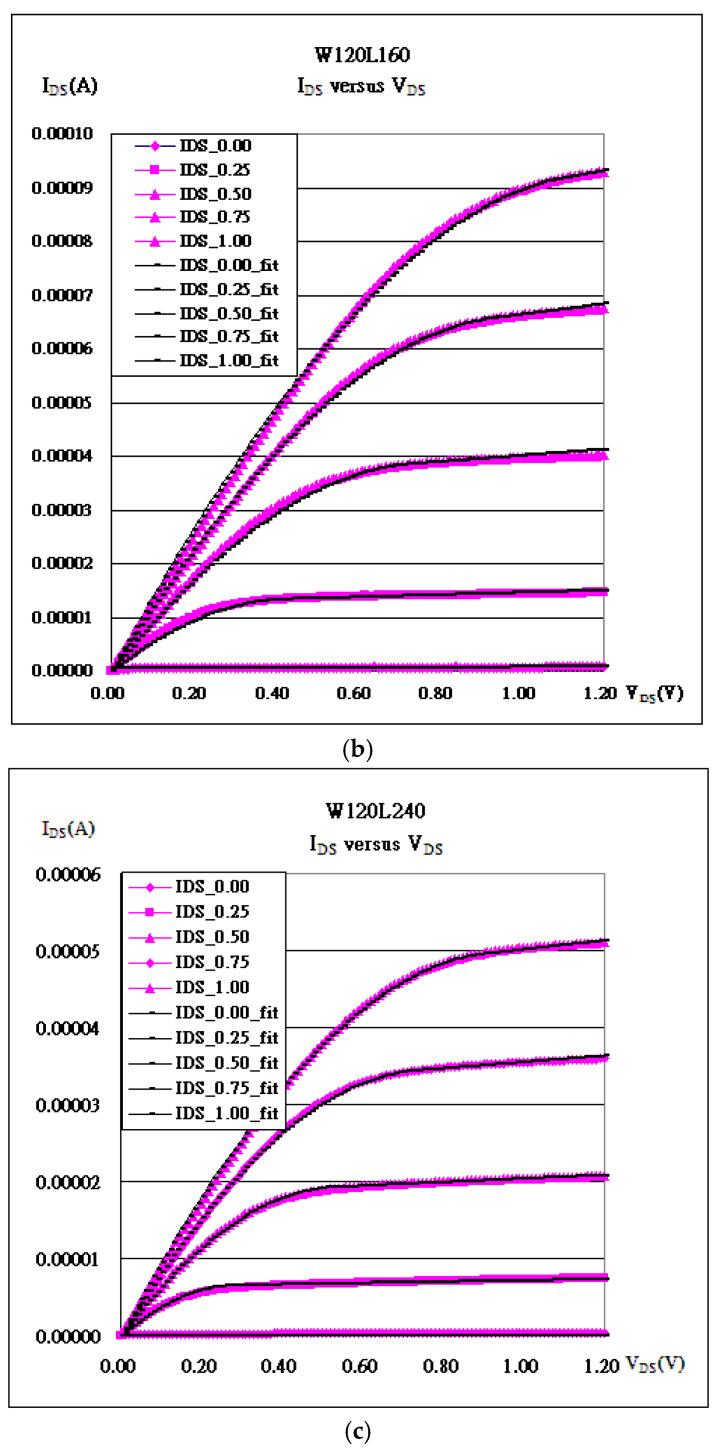
I_DS_-V_DS_ fitting of FinFET transistors with channel width 120 nm: (**a**) W120L100; (**b**) W120L160; (**c**) W120L240.

FinFET transistors with three various channel lengths are fitted with the modified conventional formula. k_N_ is extracted for analysis of mobility as in Table 1. As demonstrated in Figure 4, the kink effect is also added to make the fitting as perfect as possible. The fitting procedures include (1) lambda (λ) for the slope, (2) Vth for the turning point at *V_DS_ =* (*V_GS_* − *Vth*), and (3) k_N_ for current level adjustment. The above fitting can be concluded in a peak-like δ-V_DS_ graph, which may be flattened by the kink effect.

### 2.2. Extraction of Three Parameters (k_N_, Vth, λ) at Zero-Biased V_GS_

**Figure 5:**
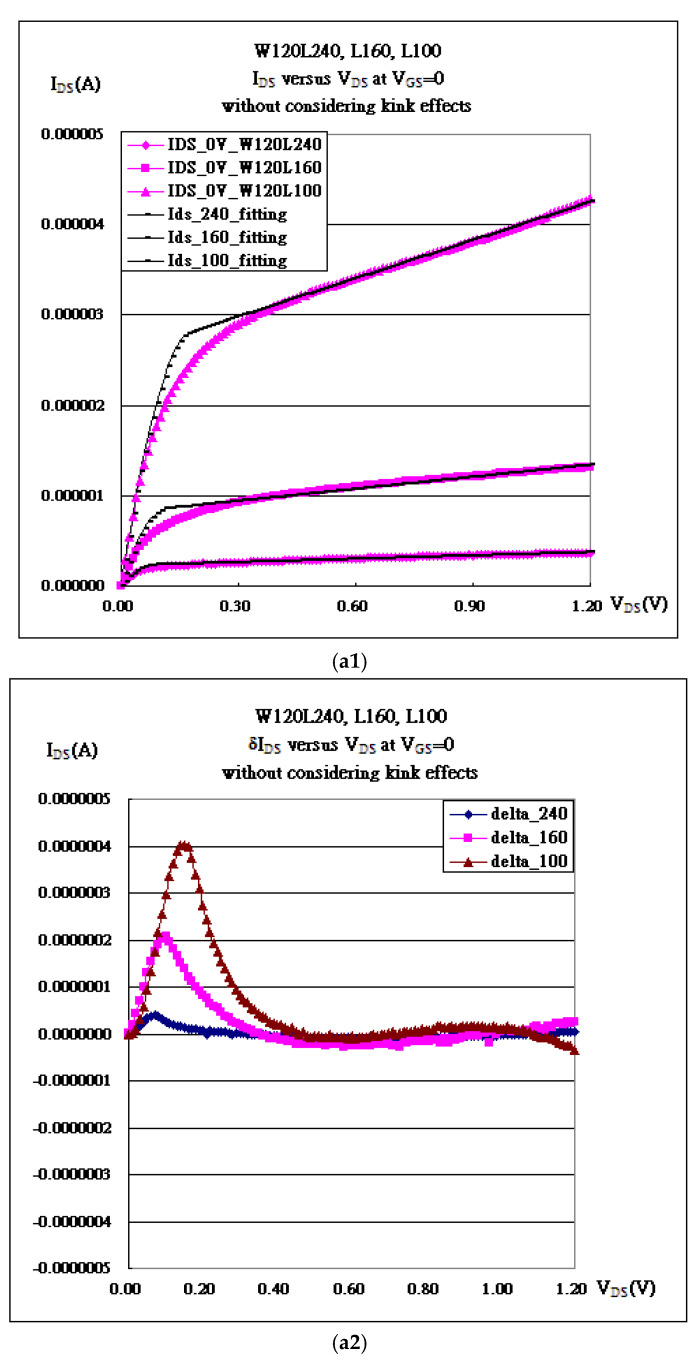

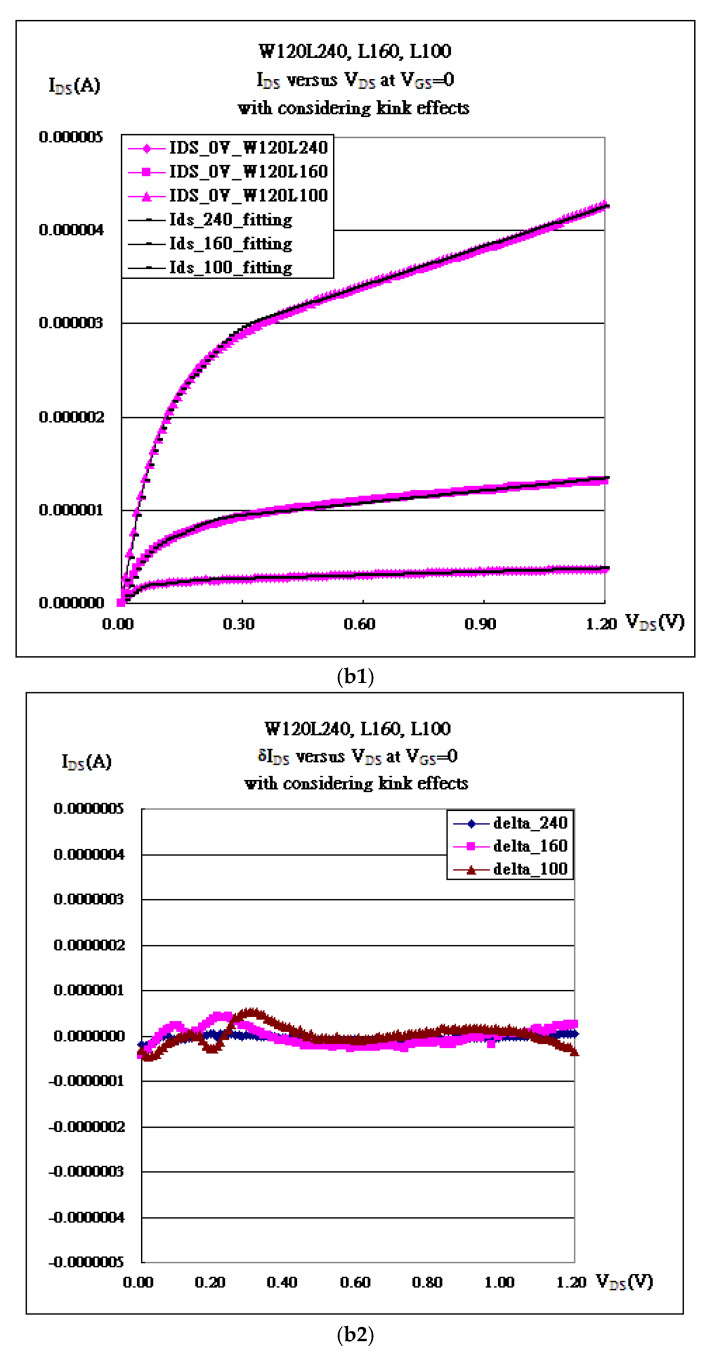
I_DS_-V_DS_ fitting of FinFET transistors with channel length 100 nm, 160 nm and 240 nm at V_GS_ = 0 V (**a1**,**a2**) with no kink effect; (**b1**,**b2**) with kink effect.

FinFET transistors with three various channel lengths are fitted with the modified conventional formula. k_N_ is extracted for analysis of mobility as in Table 1 and as demonstrated in Figure 5.

## 3. Results and Discussion

### 3.1. Leakage Current at Various V_GS_

Instead of defining I_off_, the whole characteristic curve at V_GS_ = 0 somewhat equivalently demonstrates the leakage current, which may be expressed by the term, “λV_DS_” in Equation (1). And “λV_DS_” is the ratio of leakage current (I_leakage_) to saturation current (I_o_ at V_DS_ = V_GS_ − Vth), and an equivalent r_o_ is internally inserted in between the Drain and Source as follows:

$$\lambda V_{DS}\equiv \frac{I_{leakage}}{I_{o}}=\frac{\left(V_{DS}/r_{o}\right)}{I_{o}}=\frac{V_{DS}}{I_{o}r_{o}}=\frac{V_{DS}}{\left|V_{A}\right|},$$

where VA is Early voltage.

In Table 1, the larger the channel length is, the less leaky the transistor is at non-zero V_GS_. At V_GS_ = 0, the leakage current associated with the same λ = 0.55 is not even particularly controllable because there is no depletion region (in general, directly generated from the Gate) to help block leakage across the Drain and Source. For all three different channel lengths, the same λ (i.e., the same early voltage) indicates that r_o_ was proportional to channel length with the same channel width if the mobility was the same. Actually, the corresponding equivalent internal resistances are 9.32 × 10^6^ Ohm (W120L240), 2.67 × 10^6^ Ohm (W120L160), and 8.78 × 10^5^ Ohm (W120L100).

### 3.2. Mobility Proportional to Channel Length

**Figure 6:**
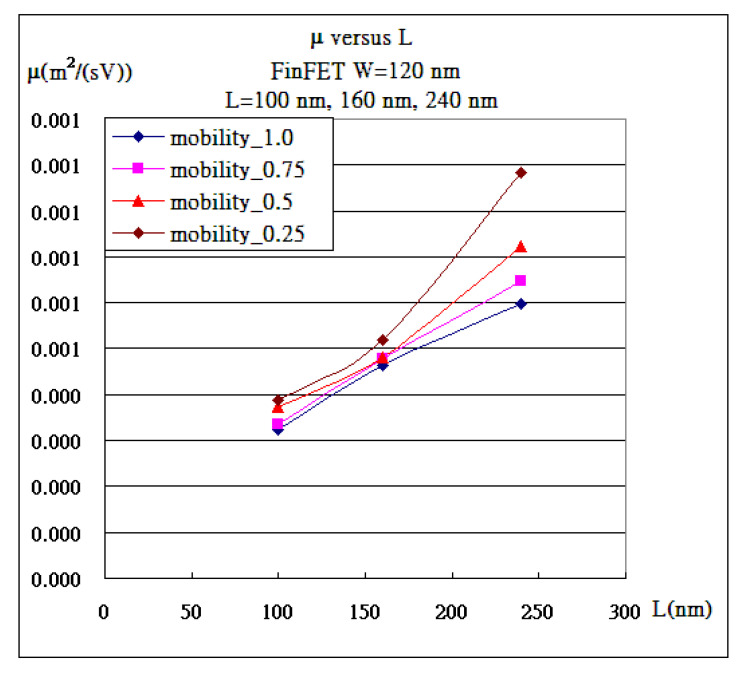
Mobility is sensitive to channel length, and mobility is linearly proportional to channel length (L) (μ/L is almost the same.)

The three FinFET transistors are all 120 nm in fin width, which at least helps prevent the issue of over etching. Actually, the aspect ratio is 9, making the effective width 19 times that of 120 nm. On the other hand, the channel length is the length that carriers move across in between the Source and Drain. The shorter the channel length is, the faster the carriers transmit the signals. Preferably, shorter channels are very competitive. But why is the mobility lower for shorter channel, such as 5 nm FinFETs or 2 nm GAAFETs? Originally, carriers are accelerated by the electric field and drift across the channel confronting viscosity obstacles from the vibrating lattice, which is presumably proportional to the power of N of the speed. Promptly, carriers reach the saturation of speed in a very short distance, which is mainly contributed to by near-zero denominators in the integration, as below. N needs setting to 1 if the speed of carriers is presumably proportional to the electric field with the mobility as the proportionality. Then mobility, μ, must be inversely proportional to the viscosity, ζ, and ζL = constant for fixed V_DS_. This means that ζ is inversely proportional to the channel length, L, at fixed V_DS_, while μ is inversely proportional to ζ. Therefore, μ is proportional to L, as shown in Figure 6. So, why is viscosity inversely proportional to the channel length? It is because of the corresponding standing sound wave at a longer channel length having a longer wavelength and lower vibration frequency, which causes less viscosity. The derivation is presented in detail as follows:

$$\begin{array}{l} F_{DS}=m_{e}a=m_{e}\frac{dv}{dl}v=-eE_{DS}+\varsigma v^{N}\\ \frac{dv}{dl}v=-\frac{e}{m_{e}L}V_{DS}+\frac{\varsigma }{m_{e}}v^{N}\\ l=\int_{0}^{v_{term}}\frac{m_{e}vdv}{-eE_{DS}+\varsigma v^{N}}\\ l=\int \frac{vdv}{-\frac{eV_{DS}}{m_{e}L}+\frac{\varsigma }{m_{e}}v^{N}}\\ -\frac{eV_{DS}}{L}+\varsigma v^{N}≅0\\ v=\sqrt[{N}]{\frac{eV_{DS}}{L\varsigma }}\overset{N=1}{\to }v=\frac{eV_{DS}}{L\varsigma }=\frac{e}{\varsigma }E_{DS}\\ \Rightarrow \left\{\begin{array}{l} \mu =\frac{e}{\varsigma }\\ L\varsigma =const,\;for\_fixed\_V_{DS} \end{array}\right. \end{array}$$

### 3.3. Mobility Inversely Proportional to E^−1/3^

As seen in Figure 2, the strong inversion layer is so narrow that the conductive carriers can only move in between the Source and Drain and squeeze in the Δx narrow aisle, which is perpendicular to the channel length. The following proof first takes Fermion statistics into account with the potential energy to verify the concentration distribution, which is substituted in the divergence of electric field (the first equation of Maxwell’s equations). The differential equation is integrated and the scope, Δx, of the strong inversion layer is discovered. There are actually two parts after the integration. One is concerning the depletion region where no free carriers exist. The other one is concerning strong inversion layers where free carriers carry signals back and forth in between the Source and Drain. The electric field due to V_GS_ depletes the channel with the boundary D beyond which E(x > D) is 0, i.e., E(x < D) = (−ep_o_/ε_Si_)(x − D) = (−ep_o_/ε_Si_)X. There is no free carrier in the depletion region and E(x) is linearly proportional to X as follows:

$$\begin{array}{l} \nabla \cdot \vec{E}=\frac{\rho }{\varepsilon_{Si}}=\frac{-ep(x)}{\varepsilon_{Si}}\\ \Rightarrow \frac{dE}{dx}=\frac{-ep_{o}}{\varepsilon_{Si}}\\ \Rightarrow E(x)=\frac{-ep_{o}}{\varepsilon_{Si}}(x-D) \end{array},$$

with boundary condition $E(D)=0for\;x\geq D$

**Figure 7:**
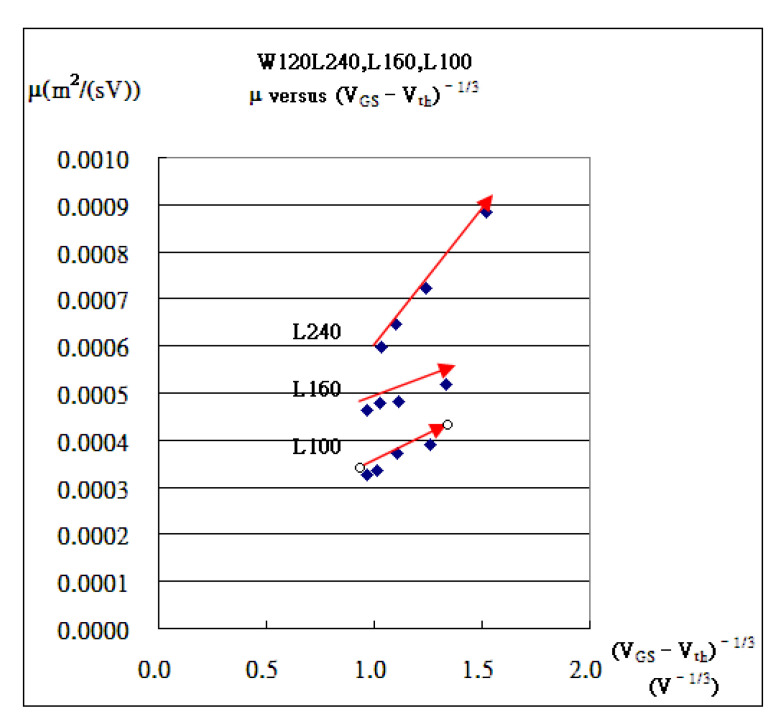
Mobility tends to be inversely proportional to E^1/3^~(V_GS_ − Vth)^1/3^ (for the collected 3 devices, W120L100, W120L160, and W120L240).

Besides this, E(x) generates both the depletion region and a free statistical carrier part, where E(x)~X^3^ (i.e., Δx = ΔX = E^(−1/3)^) addresses the uncertain region. In addition, Δp_x_ is about the same order of Δp_L_~Δ(*m_e_v*)~μ along the channel. The uncertainty principle, Δ*x*Δ*p_x_*~*E*^(1/3)^*μ*~*h*, verifies that μ is inversely proportional to Δx~E^(1/3)^, which means μ~E^(−1/3)^ (Figure 7). Physically, the stronger the electric field (V_GS_) is, the harder carriers move because E may attract carriers toward the interface to bump into the interface and result in extra friction [14].

$$\begin{array}{l} \nabla \cdot \vec{E}=\frac{\rho }{\varepsilon_{Si}}=\frac{-ep(x)}{\varepsilon_{Si}}\\ \Rightarrow \frac{dE}{dx}=\frac{-e}{\varepsilon_{Si}}(p_{o}\frac{V(x)}{0.0259})=\frac{p_{o}}{0.0259\varepsilon_{Si}}U(x)\\ \Rightarrow \frac{dE}{dx}=\frac{-p_{o}e}{0.0259\varepsilon_{Si}}[\frac{p_{o}e}{2\varepsilon_{Si}}(x-D{)}^{2}]\\ \Rightarrow \frac{dE}{dX}=\frac{-p_{o}e}{0.0259\varepsilon_{Si}}[\frac{p_{o}e}{2\varepsilon_{Si}}X^{2}]\\ E(X)=\frac{-p_{o}e}{0.0259\varepsilon_{Si}}\frac{p_{o}e}{6\varepsilon_{Si}}X^{3}\\ with\;X\equiv x-D\\ \Rightarrow \Delta x_{Str}=\Delta X=(\zeta E{)}^{\frac{1}{3}} \end{array}$$

$$\begin{array}{l} \left\{\begin{array}{l} \Delta x_{\Delta p\_fixed}=(\zeta E{)}^{\frac{1}{3}}\\ \Delta p_{\Delta x\_fixed}=m_{e}\Delta v=\gamma \mu \end{array}\right.\\ \Delta x\Delta p≅h\Rightarrow \mu E^{\frac{1}{3}}=const \end{array}$$

### 3.4. Boundary Problems with the Zero-Biased V_GS_

**Figure 8:**
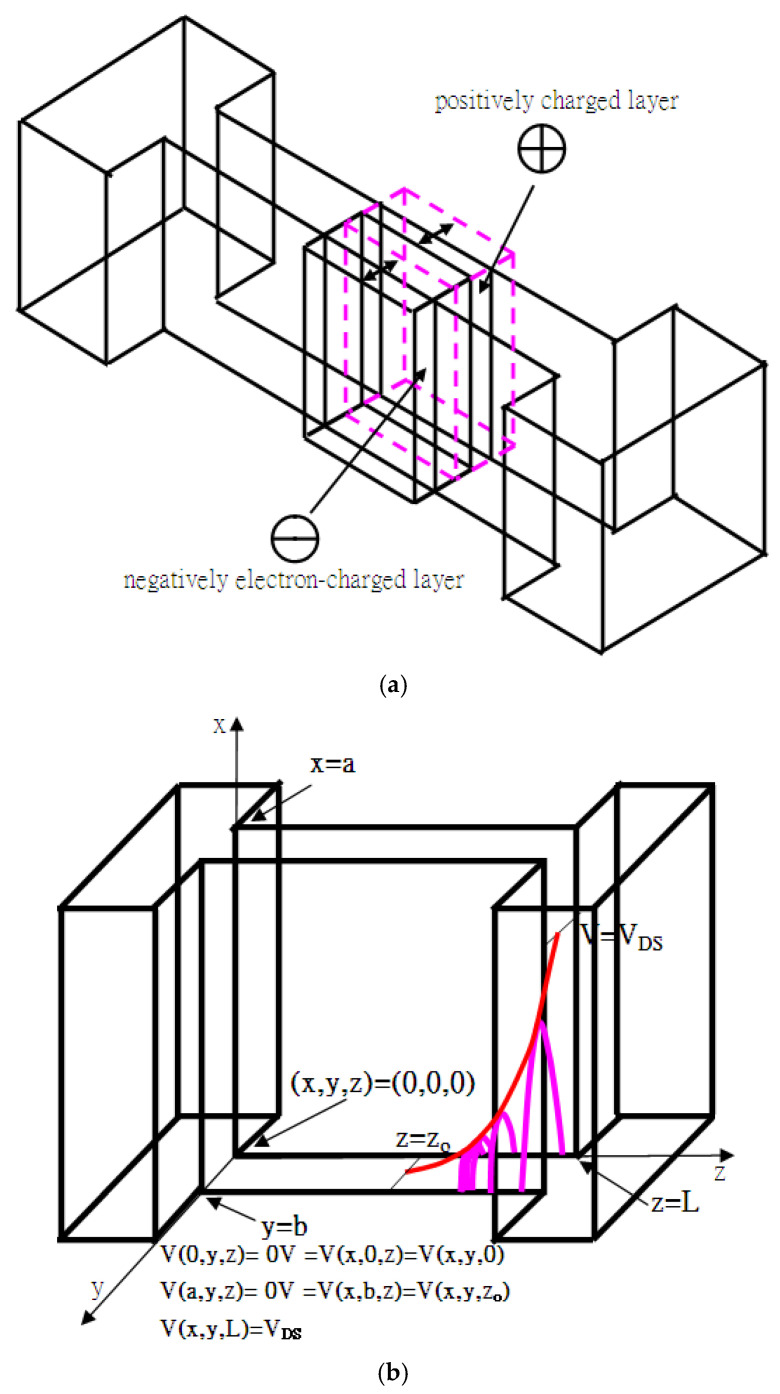
(**a**) Laplace’s differential equation giving the possibility of plasma oscillation; (**b**) the asymptotical solution of the combination of Laplace’s differential equation and Poisson’s differential equation (it is a naïve expression of potential function because the solution is a kind of field that is spatially at the same level).

Surprisingly, the depletion region and strong inversion layer might have appeared even though no Gate bias was applied. It is speculated that there must have existed a mechanism making it so. V_DS_ may be applied to the Drain either suddenly or gradually from zero to non-zero V_DS_. Therefore, for both fields, it is mandatory to introduce the boundary value problems. One is Laplace’s differential equation as in Equation (3), where no depletion region is taken into account, and the other is Poisson’s differential equation, as in Equation (4). Along the *x*-axis or *y*-axis, as in Figure 8, there exist complete sets to fit the boundary value problem. Along the *z*-axis, the solutions are trivial [15]. The pinch-off near the Drain (z = L) can be observed in Equation (4).

(1) Laplace equation:

(3) $$\begin{array}{l} \nabla^{2}V=0\\ V(x,y,z)=\sum_{m,n}V_{mn}\sin(\frac{m\pi x}{a})\sin(\frac{n\pi y}{b})\sinh(\gamma_{mn}z)\\ \left\{\begin{array}{l} (\frac{m\pi }{a}{)}^{2}+(\frac{n\pi }{b}{)}^{2}=\gamma_{mn}^{2}\\ V_{DS}=\sum_{m,n}V_{mn}\sin(\frac{m\pi x}{a})\sin(\frac{n\pi y}{b})\sinh\gamma_{mn}L \end{array}\right.\\ V_{DS}\frac{2a}{m\pi }\frac{2b}{n\pi }=V_{mn}\frac{a}{2}\frac{b}{2}\sinh\gamma_{mn}L\\ \Rightarrow V_{mn}=\frac{16V_{DS}}{mn\pi^{2}\sinh\gamma_{mn}L}\\ where\\ m\&n=positive\_odd \end{array}$$

(2) Poisson Equation:

(4) $$\begin{array}{l} \nabla^{2}V=\frac{ep}{\varepsilon_{Si}}\;or\;\nabla \cdot \vec{E}=-\frac{ep}{\varepsilon_{Si}}\;or\;\frac{dE}{dz}=-\frac{ep}{\varepsilon_{Si}}\\ E_{z}=-\frac{ep}{\varepsilon_{Si}}(z-z_{o})\begin{array}{cc} & as\;z>z_{o} \end{array}>0\\ V(x,y,z)=\sum_{m,n}V_{mn}\sin(\frac{m\pi x}{a})\sin(\frac{n\pi y}{b})(\frac{V_{DS}}{{\left(L-z_{o}\right)}^{2}}){\left(z-z_{o}\right)}^{2}\\ V_{DS}\frac{2a}{m\pi }\frac{2b}{n\pi }=V_{mn}\frac{a}{2}\frac{b}{2}V_{DS}\\ \Rightarrow \left\{\begin{array}{l} V_{mn}=\frac{16}{mn\pi^{2}}\\ z_{o}=D<L \end{array}\right. \end{array}$$

Without considering the depletion region, the p-type semiconductor is neutral and plasma oscillation may happen due to the thermal agitation of the collective electrons as shown in Figure 8a. As seen in Figure 8a, a sudden collective thin layer of electrons extensively appears on one side of the slim channel due to thermal agitation. The sinusoidal potential forms an attracting force to draw the positively or negatively charged layer back such that the oscillation collectively occurs, as derived in Equation (5). It happens even for the solution of Poisson’s equation, as shown in Figure 8b. This sudden layered charge may generate an electric field and deplete the slim channel, making it strongly inversed. In the meantime, positive/negative may switch to negative/positive according to the plasma oscillation frequency, and so on [16].

(5) $$\begin{array}{l} \nabla^{2}V=0(thin\_film\_plasma\_oscillation)\\ F_{e}=m_{e}a=eE_{x}=-e\frac{\sigma_{e}}{\varepsilon_{Si}}=-\frac{e^{2}p}{\varepsilon_{Si}}x\\ \frac{d^{2}x}{dt^{2}}=-\frac{e^{2}p}{m_{e}\varepsilon_{Si}}x=-\omega_{plasma}^{2}x\\ \omega_{plasma}=\sqrt{\frac{e^{2}p}{m_{e}\varepsilon_{Si}}}=9.88\times 10^{12}/\sec \end{array}$$

### 3.5. Threshold Voltage

The characteristic curves with negative threshold voltage behave like the traditional ones. That is, conventional formulas including the triode region and saturation region may apply in addition to Ioff or leakage current alone. For more precise fitting, the kink effect may be introduced like others. As has long been well known, initial parabolic I_DS_-V_DS_ characteristics curves concerning saturated flats are much associated with strong inversion layers, which are the result of surpassing the limits of threshold voltages. There are several conceptual steps to achieve this goal. (1) Flat-band voltage (work function difference) makes the transistor return to the starting point. The work function difference between the poly-silicon gate and the channel is *V_Flat_* = (*−*Φ*_F_*) − 0.55 = −0.991 V. (2) Conventionally, threshold voltage is mostly due to V_GS_ being larger than or equal to 2Φ*_F_* = 2(*k_B_T*/*e*)*ln*[*p*/*n_i_*] = 0.881 V, which bends down intrinsic Fermi energy to obtain a strong inversion layer. (3) With zero V_GS_, it would be impossible for channel doping, fixed oxide charge, interface charge, charge sharing effects, etc., to have to take care of at least the 2Φ*_F_* = 0.882 V, which statistically allows extra free electron carriers to move in the strong inversion layer. Even if it could, the reliability is worrying. (4) There might be another option like plasma oscillation in which the mechanism might propose the possibility of raising 2Φ*_F_* that builds up a strong inversion layer. Unfortunately, attempts to verify and test the plasma oscillation are still ongoing.

## 4. Conclusions

In this paper, (1) three 120 nm wide FinFET transistors (W120L100, W120L160, W120L240) with a channel length of 100 nm, 160 nm, and 240 nm are presented, which are well fitted by using the minimum of the root-mean-square in Equation (1). The kink effect is also demonstrated as V_GE_ is set to zero. (2) The mobility is sometimes zero such that carriers do not move even though they are in the strong inversion layer, especially in the case of Gate bias which is less than the threshold voltage. The less-than-Vth carriers in the strong inversion layer are realized to be subjected to Quantum Confinement. (3) For these moving carriers, the associated (k_N_ Vth, λ) values are listed in Table 1, and the corresponding mobility (μ) is calculated in Table 2. Mobility (μ) is then found to be inversely proportional to viscosity (ζ), which is inversely proportional to channel length (L). This means μ is proportional to L. Physically in between the Source and Drain, there should exist standing waves with wavelengths of reasonable fractions of L. The longer L is, the longer the wavelength of the standing wave is. The frequency of the standing wave is then lower, reducing the obstacles. In other words, the quantized phonons are less energetic, allowing the viscosity to decrease. (4) Mobility (μ) is proved to be proportional to E^(−1/3)^, as shown in Figure 7. By imposing the generated carriers from Fermi–Dirac statistics, p(x) is substituted into Coulomb’s Law in Maxwell’s equations. Δx is then proportional to E^(1/3)^. The uncertainty principle then helps demonstrate that the linearity in Figure 7 is true. (5) Zero-biased V_GS_ over FinFET channels makes the channel a boundary condition problem. With or without considering the depletion region, the potential in 3D has been solved, leaving the sinusoidal function on the boundary, which exerts attractive forces on these collective free carriers. Massive collective electrons move back and forth with a frequency of plasma oscillation in between two walls of the fin. The plasma frequency might be detected electrically as applied with EM waves on the IC, which could lead to resonant stimulating EM waves in the spectrum.

## Figures and Tables

**Table 1:** (k_N_, Vth, λ).

V_GS_ (V)	k_N_ (A/V^2^) (L100)	Vth (V) (L100)	λ (1/V) (L100)	k_N_ (A/V^2^) (L160)	Vth (V) (L160)	λ (1/V) (L160)	k_N_ (A/V^2^) (L240)	Vth (V) (L240)	λ (1/V) (L240)
0.00	1.60 × 10^−4^	−0.1790	0.55	1.20 × 10^−4^	−0.1160	0.55	7.00 × 10^−5^	−0.0800	0.55
0.25	1.70 × 10^−4^	−0.2498	0.31	1.42 × 10^−4^	−0.1682	0.17	1.61 × 10^−4^	−0.0302	0.13
0.50	1.63 × 10^−4^	−0.2350	0.32	1.31 × 10^−4^	−0.2227	0.17	1.31 × 10^−4^	−0.0233	0.13
0.75	1.46 × 10^−4^	−0.2088	0.32	1.31 × 10^−4^	−0.1804	0.17	1.18 × 10^−4^	−0.0200	0.13
1.00	1.42 × 10^−4^	−0.2000	0.32	1.25 × 10^−4^	−0.1065	0.17	1.09 × 10^−4^	−0.0969	0.13

**Table 2:** k_N_(A/V^2^) corresponding to μ (m^2^/Vsec) with C^(1)^ = 1.92 × 10^−2^ F/m^2^.

V_GS_	k_N_ (A/V^2^) (L100)	μ (m^2^/Vsec) (L100)	k_N_ (A/V^2^) (L160)	μ (m^2^/Vsec) (L160)	k_N_ (A/V^2^) (L240)	μ (m^2^/Vsec) (L240)
0.00	1.60 × 10^−4^	3.66 × 10^−4^	1.20 × 10^−4^	4.39 × 10^−4^	7.00 × 10^−5^	8.78 × 10^−4^
0.25	1.70 × 10^−4^	3.89 × 10^−4^	1.42 × 10^−4^	5.20 × 10^−4^	1.61 × 10^−4^	8.84 × 10^−4^
0.50	1.63 × 10^−4^	3.73 × 10^−4^	1.31 × 10^−4^	4.80 × 10^−4^	1.31 × 10^−4^	7.22 × 10^−4^
0.75	1.46 × 10^−4^	3.34 × 10^−4^	1.31 × 10^−4^	4.79 × 10^−4^	1.18 × 10^−4^	6.48 × 10^−4^
1.00	1.42 × 10^−4^	3.25 × 10^−4^	1.26 × 10^−4^	4.62 × 10^−4^	1.09 × 10^−4^	5.97 × 10^−4^

## Data Availability

No data were created, or data is unavailable due to privacy or ethical restrictions.
